# RETRACTED: Russo et al. Chamazulene-Rich *Artemisia arborescens* Essential Oils Affect the Cell Growth of Human Melanoma Cells. *Plants* 2020, *9*, 1000

**DOI:** 10.3390/plants15131953

**Published:** 2026-06-25

**Authors:** Alessandra Russo, Maurizio Bruno, Rosanna Avola, Venera Cardile, Daniela Rigano

**Affiliations:** 1 Department of Drug Sciences, University of Catania, Via S. Sofia 64, 95125 Catania, Italy; alrusso@unict.it; 2 Department of Biological, Chemical and Pharmaceutical Sciences and Technologies (STEBICEF), University of Palermo, V.le delle Scienze, Parco d’Orleans II, I-90128 Palermo, Italy; maurizio.bruno@unipa.it; 3 Centro Interdipartimentale di Ricerca “Riutilizzo Bio-Based Degli Scarti da Matrici Agroalimentari” (RIVIVE), University of Palermo, I-90128 Palermo, Italy; 4 Department of Biomedical and Biotechnological Sciences, Section of Physiology, University of Catania, Via S. Sofia, 89, 95123 Catania, Italy; rosanna.avola@unict.it (R.A.); cardile@unict.it (V.C.); 5 Department of Pharmacy, University of Naples Federico II, Via Montesano 49, I-80131 Naples, Italy

The journal retracts the article titled “Chamazulene-Rich *Artemisia arborescens* Essential Oils Affect the Cell Growth of Human Melanoma Cells” [1], cited above.

Following publication, concerns were brought to the attention of the Editorial Office regarding the integrity of images presented in this publication [1].

In accordance with the journal’s standard procedures, the Editorial Office and Editorial Board conducted an investigation that identified indications of inappropriate editing and duplication relating to Figures 5 and 6, including overlapping material previously published [2] and produced by a similar authorship group, presenting different experimental conditions. Although the authors fully cooperated with the investigation, the explanations and supporting materials provided were not deemed sufficient by the Editorial Board to resolve the identified concerns. As a consequence, the Editorial Board has lost confidence in the reliability of the reported findings and has decided to retract this publication in accordance with MDPI’s retraction policy.

This retraction was approved by the Editor-in-Chief of the *Plants* journal.

The authors have been informed of this retraction and have indicated their disagreement with this decision.
